# Co-Creating Culturally Nuanced Measures of Loneliness With Māori Elders

**DOI:** 10.1093/geroni/igaa057.2064

**Published:** 2020-12-16

**Authors:** Charles Waldegrave, Chris Cunningham, Catherine Love, Giang Nguyen

**Affiliations:** 1 Family Centre Social Policy Research Unit, Lower Hutt, Wellington, New Zealand; 2 Research Centre for Maori Health and Development, Massey University, Wellington, Wellington, New Zealand

## Abstract

The growing international evidence on the impacts of social isolation and loneliness has profound implications for positive health status and wellbeing (Holt-Lunstad et. al. 2015). This raises important questions about how we measure loneliness, particularly in extended family cultures where loneliness may be experienced differently from western more individualistic cultures. In this research, key questions around loneliness and social isolation were co-created with Māori Elders and responses were compared with a standard international loneliness scale (De Jong Gierveld Loneliness Scale) to help identify universal aspects of loneliness and Māori specific aspects. The results demonstrated significant correlations between the co-created questions and the international scale. However, they also demonstrated substantial expressions of loneliness among older Māori that are not captured by standard scales showing the need for Māori specific scales which are more inclusive of their cultural norms and can provide more precise data for constructive policy making and service provision.

